# Dispersion of adeleid oocysts by vertebrates in Gran Canaria, Spain: report and literature review

**DOI:** 10.1017/S0031182021001244

**Published:** 2021-11

**Authors:** Kevin M. Santana-Hernández, Simon L. Priestnall, David Modrý, Eligia Rodríguez-Ponce

**Affiliations:** 1 Department of Animal Pathology, Faculty of Veterinary Science, University of Las Palmas de Gran Canaria, Las Palmas de Gran Canaria, Spain; 2 Department of Pathobiology and Population Sciences, The Royal Veterinary College, Hatfield, UK; 3 Department of Parasitology and Pathology, University of Veterinary and Pharmaceutical Sciences, Brno, Czech Republic; 4 Institute of Parasitology, Biology Centre of Czech Academy of Sciences, České Budějovice, Czech Republic

**Keywords:** Ecology, insect pathology, invasive species, parasitology, protozoa

## Abstract

Within the family Adeleidae, *Adelina* spp. belong to a group of arthropod pathogens. These parasites have been reported to have a wide geographic distribution, however, there are no reports of these protists in the Canary Islands, Spain. One of the peculiarities of the life cycle of *Adelina* spp. is the participation of a predator, because fecundation and sporulation occur inside the body cavity, and so necessitate destruction of the definitive host. The involvement therefore of a ‘dispersion host’, which eats the definitive host and spreads the oocysts through its faeces, is critical for the maintenance of certain *Adelina* spp. On the island of Gran Canaria, adeleid oocysts have been found in stool samples from four animals, three California kingsnakes (*Lampropeltis californiae*), and one feral cat. These animals were part of a larger coprological study of vertebrate parasites (117 snakes, 298 cats), where pseudoparasitic elements were also recorded. *L. californiae* and feral cats are invasive species which are widespread across the island and this novel finding of *Adelina* spp. oocysts in their faeces suggests that they could also serve as potential sentinel species for arthropod parasites.

## Graphical abstract

**Figure:**
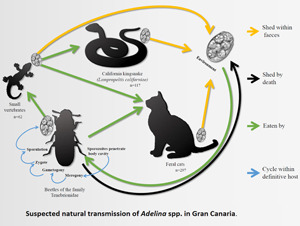

## Introduction

*Adelina* spp. (Apicomplexa: Adeleroina: Adeleidae) are parasitic protists of invertebrates, reported to have a worldwide distribution (Berto *et al.,* 2010). However, knowledge of the diversity of these protists is rather limited, particularly when compared to the diversity of their hosts. In the Canary Islands, an autonomous region of Spain located in the Macaronesian North Atlantic, there are no reports of *Adelina* spp. On the Iberian Peninsula, insect-related Adeleids have been observed as intra-abdominal oocysts in permanent mounts of sand flies (Morillas-Marquez *et al.,* 1983; Martinez-Ortega and Conesa-Gallego, 1987). These have only been identified to genus level which is understandable considering the large overlap in morphological parameters which exists between most of the described species (Purrini, 1984; Berto *et al.,* 2010).

The pathogenicity of these protozoa has not been studied extensively in natural invertebrate communities, however, their capacity to contribute to species competition, behavioural and colour changes, paralysis, darkening of internal organs and ultimately as a cause of death, have been demonstrated (Table 1). Thus, in addition to their likely natural role in population regulation, there may be a role for *Adelina* spp. as a means of biological pest control in farming (Yarwood, 1937; Park and Frank, 1950; Weisner, 1964; Purrini, 1984; El-Sufty and Boraei, 1989).

**Table 1.** Recorded pathological effects of *Adelina* spp. on arthropod species around the world under laboratory or natural (Lab/Nat) conditions

Effect	Parasite	Host Order	Host family	*Host Spp*	Instar	Country	Lab/nat	Reference
Behavioural changes	*A. hypera*	Coleoptera	Curculionidae	*Hypera brunneipennis*	Larvae	Egypt	Lab	El-Sufty and Boraei (1989)
	*A. tribolii*	Coleoptera	Tenebrionidae	*Tribolium ferrugineum*	Larvae	Cambridge, UK	Lab	Bhatia (1937)
Colour changes	*A. hypera*	Coleoptera	Curculionidae	*Hypera brunneipennis*	Larvae	Egypt	Lab	El-Sufty and Boraei (1989)
	*A. collembolae*	Collembola	Neanuridae	*Neanura muscorum*	Adults	Germany	Nat	Purrini (1984)
Dark-brown spots in infected tissue	*A. hypera*	Coleoptera	Curculionidae	*Hypera brunneipennis*	Larvae	Egypt	Lab	El-Sufty and Boraei (1989)
	*A. cryptocerci*	Blattodea	Cryptocercidae	*Cryptocercus punctulatus*	Adults	Oregon, USA	Lab	Yarwood (1938)
	*A. collembolae*	Collembola	Neanuridae	*Neanura muscorum*	Adults	Germany	Nat	Purrini (1984)
Death	*Adelina* sp.	Coleoptera	Curculionidae	*Hypera brunneipennis*	Adults	Egypt	Nat	Merritt *et al.* (1975)
	*Adelina* sp.	Coleoptera	Curculionidae	*Hypera brunneipennis*	Larvae	Egypt	Nat	Merritt *et al.* (1975)
	*Adelina* sp.	Coleoptera	Curculionidae	*Hypera brunneipennis*	Larvae	Egypt	Nat	El-sufty and Boraei (1986)
	*A. hypera*	Coleoptera	Curculionidae	*Hypera brunneipennis*	Larvae	Egypt	Lab	El-sufty and Boraei (1989)
	*A. hypera*	Coleoptera	Curculionidae	*Hypera brunneipennis*	Cocoon	Egypt	Lab	El-sufty and Boraei (1989)
	*A. cryptocerci*	Blattodea	Cryptocercidae	*Cryptocercus punctulatus*	Adults	Oregon, USA	Lab	Yarwood (1937)
	*A. tribolii*	Coleoptera	Tenebrionidae	*Tribolium castaneum*	Larvae	Chicago, USA	Lab	Park and Frank (1950)
	*A. tribolii*	Coleoptera	Tenebrionidae	*Tribolium castaneum*	Pupae	Chicago, USA	Lab	Park and Frank (1950)
	*A. tribolii*	Coleoptera	Tenebrionidae	*Tribolium confusum*	Larvae	Chicago, USA	Lab	Park and Frank (1950)
	*A. tribolii*	Coleoptera	Tenebrionidae	*Tribolium confusum*	Pupae	Chicago, USA	Lab	Park and Frank (1950)
	*A. tribolii*	Coleoptera	Tenebrionidae	*Tribolium ferrugineum*	Larvae	Cambridge, UK	Lab	Bhatia (1937)
Population regulation	*A. tribolii*	Coleoptera	Tenebrionidae	*Tribolium castaneum*	–	Chicago, USA	Lab	Park and Frank (1950)

*Adelina* spp. are currently divided into two lineages; one group is found in the body cavity, while the second includes gut parasites. Classically, the genus *Adelina* (body cavity parasites) was erected from *Adelea* spp. (intestinal parasites), with differentiation of the two genera based on morphology of the sporocysts, which are spherical and discoidal, respectively (Yarwood, 1937). Based on these morphological features, several species from *Adelea* and *Klossia* were reclassified within the genus *Adelina*. However, with the exception of *Adelina dimidiata* and *A. schellacki,* which infect myriapods, all *Adelina* spp. are body cavity parasites (Purrini, 1984). Few molecular genetic studies have been undertaken in this genus, however comparing available sequences from NCBI (accession numbers in brackets), the difference of 4.3% between *A. dimidiata* (DQ096835.1) and *Adelina grylli* (body cavity) (DQ096836.2) is greater than other apicomplexans such as *Cystoisospora canis* (KT184368.1) compared with *Toxoplasma gondii* (2.2%, V03070.1;KX008033.1), *Neospora caninum* (1.9%, L24380.1) or *Besnoitia* spp. (*B. darlingi* (1.8%) MF872603.1; *B. besnoiti* (1.5%) XR_003828658.1). Further research is clearly needed to refine the current taxonomical status of these species and thus the intestinal infecting *Adelina* species are not considered further in this review.

The life cycle of *Adelina* spp. occurs inside the arthropod body cavity, with sporozoites piercing the gut to access the coelom (Merritt *et al.,* 1975). Asexual division takes place, forming two generations of merogonies (as described for *A. cryptocerci)* followed, after release of the merozoites into fatty tissue, by sexual reproduction of gametoblasts (Yarwood, 1937). These macro and microgametoblasts fuse and develop into a zygote, which finally forms a sporont (Yarwood, 1937; Park and Frank, 1950; Ghosh *et al.,* 2000). Sporulation generally occurs within the fat bodies. As the infection spreads, the body tries to encapsulate the oocysts within tissue, to isolate them, and these appear as dark aggregates (Park and Frank, 1950; El-Sufty and Boraei, 1989). Finally, the adeleids begin to occupy the majority of the coelom and the rest of organs including muscles, resulting in death of the insect (Bhatia, 1937; Park and Frank, 1950; El-Sufty and Boraei, 1989). Other authors report secondary infections with gut bacteria as a cause of death in invertebrates, after penetration through the gut wall by the coccidia (Merritt *et al.,* 1975).

To infect other hosts, the oocysts must be released to the environment and then be ingested by other invertebrates. This can happen by cannibalism or through a ‘dispersion host’ (Sautet, 1930; Butaeva, 1996; De Quadros *et al.,* 2017). A dispersion host is typically a vertebrate predator which ingests an invertebrate whose tissues contain *Adelina* oocysts, and which are then released into its digestive tract and excreted. This phenomenon has been observed in several vertebrate species (reptiles, amphibians, birds and mammals), in which the parasite-infected invertebrates form part of their diet (Barnard *et al.,* 1974; Berto *et al.,* 2008; Lopes *et al.,* 2013; De Quadros *et al.,* 2017).

The Canary Islands are an archipelago composed by eight islands and five islets in Macaronesia. Despite their small size (7447 km^2^), the Canaries are home to one of the largest number of endemic species in the temperate regions globally (Machado, 1998). Among the varied landscapes of the islands, which are considered ‘hot-spots’ of biodiversity, the laurel forests are particularly unique, found only in Macaronesia (Machado, 1998). Even considering their small size, there are between 2 and 5 isoclimatic zones, depending on the island, with four in the case of Gran Canaria: dry desert, dry steppe, temperate mild and temperate cold (Rodríguez-Ponce *et al.,* 1995).

On Gran Canaria, 5872 species of flora and fauna have been recorded to date, of which 22.7% are considered endemic. Arthropods comprise the largest and most diverse group with 3190 species recorded to date, of which 32.1% are endemic to the island (Arechavaleta *et al.,* 2010). Although arthropods constitute more than half the total species described on the island, there is a total dearth of knowledge of their coccidian parasites or their potential role in the regulation of arthropod populations within the islands. Moreover, considering the introduction of foreign parasitic species into the islands by exotic arthropods [612 introduced species and 66 invasive species. (Arechavaleta *et al.,* 2010)], an evaluation of current invertebrate parasites present on the island is much needed.

This study aims to contribute to baseline data for studies on invertebrate parasites in Macaronesia, their dissemination hosts as well as documenting the oocysts found.

## Materials and methods

Between 2016 and 2019, faecal samples from various vertebrate animal species from Gran Canaria were analysed at the Laboratory of Parasitology, Faculty of Veterinary Sciences of the University of Las Palmas de Gran Canaria.

Faecal samples from cats were obtained from live animals during a larger study of feral cat colonies from across the island and donated from neutering release campaigns. For the remaining animals, the faeces were collected during *post-mortem* examination of fresh or frozen carcasses. The animals were obtained from the Tafira Wildlife Recovery Centre (naturally dead hedgehogs and birds) or Gestion y Planeamiento Territorial y Medioambiental (GesPlan) who conduct the eradication programme of invasive California kingsnakes (*Lampropeltis californiae*) in Gran Canaria. The samples from dogs were obtained during *post-mortem* examination of animals from the local animal shelter (Albergue insular de animales, Arucas) during practical classes in the Veterinary Faculty.

For species others than dogs and cats, all the collected faeces were used for concentration methods. For small amounts of sample, a minimum quantity of 0.5 mL of faeces were placed in each of three microcentrifuge tubes for processing. Samples with less than 0.5m L were discarded. For cats and dogs an average of 1.5 ***g*** of faeces were used for each concentration test. All faecal samples were tested for parasites using flotation in saturated sodium chloride solution (density 1.2 g mL^−1^), zinc sulphate centrifugal flotation (density 1.18 g mL^−1^) and formol-ether concentration method (7 parts of 10% formalin, 3 parts of pure diethyl-ether) (Willis, 1921; Faust *et al.,* 1938; Zajac and Conboy, 2012). Proper parasites and pseudoparasites were recorded.

The identification was carried by using the available references for pseudoparasitic elements in vertebrate faeces (Parker and Duszynski, 1986; Berto *et al.,* 2008; Lopes *et al.,* 2013; De Quadros *et al.,* 2017).

From each positive sample, oocysts were measured using a calibrated microscope (Leitz Laborlux S).

## Results

In all, 476 faecal samples from 298 feral cats, 117 California kingsnakes, 10 Algerian hedgehogs (*Atelerix algirus caniculus*), 15 feral dogs and 36 birds from seven species were examined. Of these birds, many were species endemic to Macaronesia (M) or subspecies endemic to the Canary Islands (C) and included 10 *Turdus merula,* 9 *Falco tinnunculus canariensis* (C), 8 *Asio otus canariensis* (C), 3 *Passer hispaniolensis,* 3 *Serinus canaria* (M), 2 *Apus unicolor* (M) and 1 *Gallinula chloropus*.

Of the 476 samples, just four contained round to slightly ellipsoidal oocysts containing more than 4 (6–16) round sporocysts, consistent with the definition of the genus *Adelina*. These positive samples were from one cat, from the municipality of La Aldea de San Nicolás, in the west of the island; and three snakes from the municipality of Telde in the east giving a total *Adelina* spp. oocyst prevalence of 0.8% (4/476) across all samples, and 0.3% (1/298) and 2.6% (3/117) of feral cat and snake samples respectively. Measurements of oocysts and sporocysts in from each species are presented in Table 2 and compared with the other *Adelina* species described in the literature (Purrini, 1984).

**Table 2.** Measurements of the stages of the parasite are given [meront (**M**), macrogametocyte (**Ma**), microgametocyte (**Mi**), and oocyst (**O**)], to summarize and facilitate the identification of future *Adelina* spp. in histological sections, fresh invertebrate tissues or as pseudoparasites in faeces

Parasite	Host	Tissue	Measurements in micrometres					NS	Author
			M	Ma	Mi	O	S		
*A. acarinae*	*Nothrus silvestris*	Body cavity	–	–	–	15–25	7–7.5	8–12	Purrini (1984)
*A. castana*	*Tribolium castaneum*	Body cavity	18–30 × 13–18	13–33 × 6.5–30	8.5–11.5 × 4–10	29.3 × 25.4	8.2	4–12	Ghosh *et al.* (2000)
*A. collembolae*	*Neanura muscorum*	Body cavity	–	–	–	40	7.5–8	24	Purrini (1984)
*A. cryptocerci*	*Cryptocercus punctulatus*	Different tissues	11 × 20	20 × 51	2.5–3	46–51 × 24–28	10–12	5–21	Yarwood (1937); Purrini (1984)
*A. deronis*	*Dero limosa*	Body cavity	25	19 × 17	7–9	17–21	9	8–16	Hauschka and Pennypacker (1942); Purrini (1984)
*A. grylli*	*Gryllus bimaculatus*	Body cavity	25.6 × 16.4	24.5 × 18.	3.5 × 2.45	32.5–36.3 × 24.7–30.1	9.9–13.3	4–22	Butaeva (1996)
*A. melolonthae*	*Melolontha melolontha*	Body cavity	18–22 × 11–14	30–50	10–11 × 6–7	30–35	11	6–14	Tuzet *et al.* (1965); Purrini (1984)
*A. octospora*	*Slavina appendiculata*	Body cavity	18–20	23 × 20	15 × 6	19–20	5–9	8	Hesse (1911); Purrini (1984)
*A. palori*	*Palorus ratzeburgii*	Body cavity	16.5–21.5 × 8–15	15–30 × 10–21	6–8	30.3 × 24.6	8	4–12	Ghosh *et al.* (2000)
*A. picei*	*Alphitobius picetus*	Body cavity	18–25 × 11.5–16.5	21–31 × 13–25	6.5–10	33.9 × 29.9	8.5	8–18	Ghosh *et al.* (2000)
*A. sericesthis*	*Sericesthis pruinose*	Body cavity	15–20 × 12–16	30–40	–	30–40	12–15	4–8	Weiser and Beard (1959); Purrini (1984)
*A. tenebrionis*	*Tenebrio molitor*	Body cavity	10–16	25	10	–	10–12	2–12	Sautet (1930); Purrini (1984)
*A. transita*	*Embia solieri*	Body cavity	30	30–40	8	30–40	10–11	6–20	Léger (1904); Purrini (1984)
*A. tribolii*	*Tribolium div.* sp.	Different tissues	15–30 × 6–20	21–49 × 16–33	8–15	26–50 × 22–36	10 × 4	2–24	Bhatia (1937) Purrini (1984)
*A. zonula*	*Blaps mortisaga*	Fat body	15–27 × 2–15	30–40	2–4 × 8–11	–	–	8	Morrof (1907); Purrini, (1984)
*A. akidum*	*Olocrates abbreviatus*	Body cavity	–	–	–	30–40	10	12–20	Léger (1900); Purrini (1984)
*Adelina* sp. ‘*picei*-like’	Cat faeces	–	–	–	–	32–33 × 28–30	8–10	6–8	This paper
*Adelina* sp. ‘*tribolii*-like’	Snake faeces 1 and 3	–	–	–	–	39–41 × 28–31	10–13	6–9	This paper
*Adelina* sp. ‘*tribolii*-like’	Snake faeces 2	–	–	–	–	52–53 × 34–35	10–11	14–16	This paper

*Adelina* spp. described, but thus far un-named, have not been considered. All the measurements are in micrometres. S, sporocyst; NS, number of sporocysts. In the author column the first one is the original description, authors in brackets are the source of the description represented in this table. If only an author in brackets is cited, represent also the original description.

**Fig. 1.**
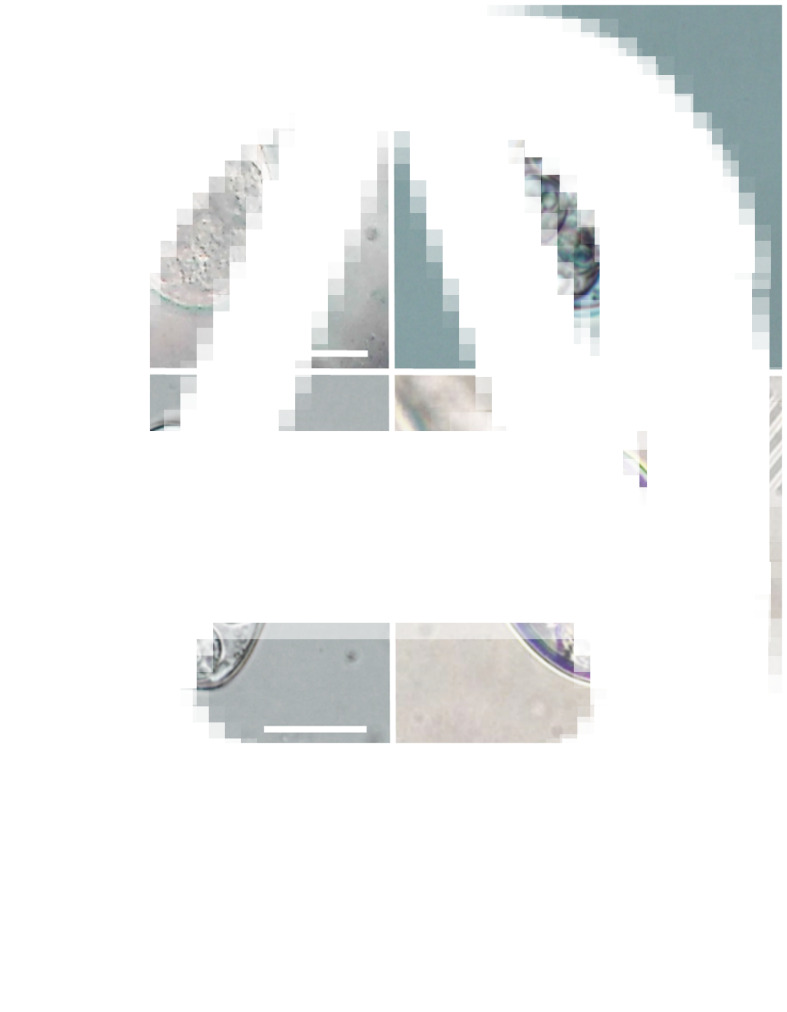
Photomicrographs of sporulated *Adelina* spp. oocysts. (A) *A. picei* from a feral cat. (B) *A. tribolii* from snake 1. (C**)** *A. tribolii* from snake 2. (D) *A. tribolii* from snake 3. Scale bars = 20 *μ*m.

Based on the size of the oocysts and sporocysts, the coccidia in the cat faeces resembled *Adelina picei* (two oocysts) (Fig. 1A), but the number of sporocysts found in these specimens was 6–8, while that described for *A. picei* is 8–18.

The coccidia from snake no. 1 (three oocysts) (Fig. 1B), were considered to be *Adelina tribolii-*like species, as the measurements and morphology (41 × 28–29 *μ*m oocysts, slightly ellipsoidal 11 × 10–11 *μ*m sporocysts, 8–9 sporocysts per oocyst) fell within the ranges of *A. tribolii* [26–50 × 22–36 *μ*m oocysts, round sporocysts 10.4 *μ*m and 2–24 sporocysts per oocyst (Purrini, 1984)]. In the faeces from snake no. 2 (two oocysts) (Fig. 1C), the coccidia most closely resembled *A. tribolii* based on the size of the oocysts and the number of sporocysts. Finally, the coccidia found in the faeces of snake no. 3 (two oocysts) (Fig. 1D) are possibly the same species as in snake no. 1 i.e. *A. tribolii*-like oocysts, but with slightly bigger sporocysts.

## Discussion

In a diagnostic laboratory, pseudoparasitic elements, as well as pollen grains, fungal spores and yeasts, dust mite eggs and even fly larvae are usually present in faecal samples at the time of analysis. With experience, the technician can distinguish what is and what is not a parasitic element. However, in the case of carnivorous animals these pseudoparasitic elements could be parasites of their prey species. Frequently these prey parasites are disrupted and may appear ‘dead’, but in the case of *Adelina* the eggs survive inside the bowel of the predator (dispersion host) and are disseminated to the environment with the faeces, in the same way ingested plant seeds would also be dispersed.

The results of this study indicate the presence of at least two species of *Adelina* resembling *A. tribolii* and *A. picei* on the island of Gran Canaria. However, morphological measures of the oocysts are close to several reported species, but with potentially important differences in sporocyst numbers (Table 2). This fact may be important from the perspective of the identification of very similar species by molecular methods, considering the huge variation in *A. tribolii* sporocysts (from 2 to 24). This variation could be also explained by the process of sporulation, with two sporocysts being erroneously reported as mature oocysts, instead of 24, or the presence of several cryptic species. In addition, the lack of further ecological, morphological and molecular data from the actual definitive host, leave the speciation just presumptive at this stage.

California kingsnakes, unlike cats, are not known to eat invertebrates and thus the presence of adeleids in the faeces of a non-insectivorous snake could be explained through their regular prey on Gran Canaria: the Gran Canaria giant lizard (*Gallotia stehlini*), geckos (*Tarentola boettgeri*), skinks (*Chalcides sexlineatus*) and rodents (Monzón-Argüello *et al.,* 2015). These prey species usually consume arthropods and thus the oocysts may have originated from invertebrates within their gastrointestinal tract. In support of this theory is the finding, in the snake faeces, of other parasites from these prey reptile species such as eggshells of Pharyngodonidae oxiurids.

Despite all species in this study having a diet which includes insects, neither species of *Adelina* spp. was found. A possible explanation, given the low prevalence obtained from snakes and cats, could be the sample size of each species, as well as the scarcity of faeces in small animals. Furthermore, the accurate diet composition of the other species of the study could also influence the species of *Adelina* to be found e.g. swifts (*Apus* spp.) prey on tiny flying insects caught on the wing which may not contain *Adelina* spp.. Previous studies on wild invertebrates demonstrate a prevalence of *Adelina* spp. between 3 and 27% (Merritt *et al.,* 1975; El-Sufty and Boraei, 1986, 1989). What is not clear is if the low prevalence studies can be explained by selection failure of the sampled arthropods, due to death of infected immature stages. Considering the wide prevalence variation reported in other studies, it is not clear if the low figure of 0.8% in this study, is truly representative of the overall prevalence of *Adelina* in Gran Canaria. These two vertebrate species (cats and snakes) could amplify the number of oocysts in faeces by consuming more prey such as geckoes, serving as sentinel species for *Adelina* spp. surveys. Further studies are required to more accurately determine the prevalence of *Adelina* within definitive and other dispersion hosts.

Although data are scarce, Adeleid coccidia could be considered important ecosystem ‘regulators’, causing death of various arthropod species (Table 1). Under laboratory conditions, 20% fewer larval stages are reported *vs* non-infected insects, demonstrating how insect populations, can be influenced by these parasites (Park and Frank, 1950). Insects which are resistant to *Adelina* spp. have a significant selective advantage over those which are non-resistant (Park and Frank, 1950; Lange and Lord, 2012). Without the selective pressure of the parasite, the non-resistant insects dominate over the resistant ones.

The presence of *Adelina* spp. in stool samples from vertebrates is important from an ecological point of view, as digestion by vertebrates is required to release the oocysts from the invertebrate tissues, and disseminate within their faeces (Parker and Duszynski, 1986; De Quadros *et al.,* 2017). This has been widely studied in other parts of the world with Adeleorid coccidia demonstrated in vertebrate faeces as pseudoparasites (Parker and Duszynski, 1986; Berto *et al.,* 2008; Lopes *et al.,* 2013; De Quadros *et al.,* 2017). Indeed, a genus of coccidia (*Pythonella* spp.) was erroneously described as a reptile parasite when it is actually a pseudoparasite (Kawazoe and Gouvêa, 1999; Ghimire, 2010).

Dispersion hosts, on occasion, travel long distances or even, in the case of migratory birds, may move from one country or region to another, disseminating their parasites to their new habitat. This phenomenon has been widely demonstrated in ticks, with tick-borne diseases being carried from one country to another (Hasle, 2013). Furthermore, novel parasites introduced by these dispersion hosts or by exotic/invasive invertebrates may cause more significant disease in naïve invertebrate hosts than the natural infected host populations (Kelehear and Jones, 2010; Bacela-Spychalska *et al.,* 2012; Martín-Torrijos *et al.,* 2017). However, host specificity and thus the real impact of *Adelina* spp. in natural invertebrate populations, compared with laboratory populations, is not currently understood. Neither co-invasion nor host switch in natural insect populations infected with *Adelina* spp. has been reported in the literature, thus, further research is needed. Indeed, Gran Canaria, with its huge invertebrate diversity could be considered an ideal model island system to study this and other invertebrate parasites, starting with morphological and molecular surveys, and promotion of conservation programmes.

In general terms, coccidian parasites, including *Adelina* spp., are very host specific, affecting mostly animals from the same genus. *Adelina tribolii* has been described in three species of flour beetles (*Tribolium* spp.) (Table 1) (Park and Frank, 1950), a genus of beetle from the family Tenebrionidae. Based on this, *A. tribolii-like* records from Gran Canaria are most-likely parasites of a *Tribolium* sp., possibly the invasive species red flour beetle (*T. castaneum*) or confused flour beetle (*T. confusum*) which are the only known species recorded on the island. The other putative species recorded in this study, *Adelina picei* has been reported parasitizing *Alphitobius* sp., another tenebrionid beetle. Considering host specificity related to the genus of the host, for *Adelina picei* another two beetle species could be suitable hosts in Gran Canaria: the introduced lesser mealworm (*A. diaperinus*) and the black fungus beetle (*A. laevigatus)*.

The definitive host species of the *Adelina* pseudoparasites remains unknown, however cats are known to consume Tenebrionid beetles often in feral life, unlike *L. californiae* (Medina and Nogales, 2009; Monzón-Argüello *et al.,* 2015; Gallo-Barneto *et al.,* 2016). Based on this data, *Adelina* could be present in Tenebrionids, of which several species are endemic and endangered (Arechavaleta *et al.,* 2010). Further sampling would be needed, in conjunction with molecular work, to address the accurate epidemiology of this parasite in Gran Canaria and other parts of the world.

## Conclusions

Despite a low prevalence, these findings constitute the first baseline data for invertebrate pathology studies in the Canary Islands. Further epidemiological research on invertebrate parasites in these islands would be necessary to determine the invertebrate hosts, native or exotic, and the real epidemiological importance of insectivorous animals in the life cycle of *Adelina* spp. The further understanding of the role of this protozoan in invertebrate population dynamics is particularly important in an island setting where the vast majority of fauna is native/endemic and/or endangered. The Canaries, and other similar islands, could be utilized as model systems for arthropod parasites. Using morphological measures, the oocysts described here are close to several reported species, but with potentially important differences in sporocyst numbers. Further material should be studied to determine its accurate taxonomical status, considering the morphological variability of *A. tribolii*. With the appropriate molecular sampling of Adeleids within invertebrates, the vertebrate species studied here could be useful as sentinels for further research on *Adelina* spp. in the Canary Islands and further afield.
